# Association Between Dietary Patterns, Weight Loss, and Handgrip Strength Among Qatari Adults with a History of Bariatric Surgery: Results from the Qatar Biobank Study

**DOI:** 10.3390/nu18091411

**Published:** 2026-04-29

**Authors:** Shada Almaket, Gana Hissain, Salma Mehrez, Joyce Moawad, Zumin Shi

**Affiliations:** Department of Nutrition Sciences, College of Health Sciences, Qatar University, Doha P.O. Box 2713, Qatar; sa2005103@student.qu.edu.qa (S.A.); gh2104059@student.qu.edu.qa (G.H.); sm2104126@student.qu.edu.qa (S.M.); jmoawad@qu.edu.qa (J.M.)

**Keywords:** dietary patterns, bariatric surgery, handgrip strength, muscle strength, weight loss, Qatar biobank study

## Abstract

**Background/Objectives:** This study examines cross-sectional associations between dietary patterns, weight loss, and handgrip strength (HGS) among adults with a history of bariatric surgery. **Methods:** We analyzed data of 1888 adults (62.3% women; mean age 38.8 years) who attended the Qatar Biobank study. Dietary patterns were identified using factor analysis of data from a food frequency questionnaire. HGS was measured using dynamometry, and relative HGS (RHGS) was calculated as HGS/BMI. **Results:** The mean weight loss after bariatric surgery was 27.6 kg (23.4%), and the mean HGS was 30.1 (SD 11.2) kg. The mean duration after bariatric surgery was 3.6 years. Greater weight loss was associated with lower HGS (Q4 vs. Q1: −1.29 (95%CI −2.26 to −0.33)) but higher RHGS (Q4 vs. Q1: 0.10 (0.06 to 0.13)). Higher adherence to a “prudent diet” with high intake of fruits and vegetables was associated with stronger HGS (Q4 vs Q1: 1.07 (0.18 to 1.96)). In contrast, a “traditional diet” (high intake of mixed dishes, e.g., biryani, croissants, zaatar fatayer, lasagna, white rice, and Arabic bread) was inversely associated with HGS (Q4 vs. Q1: −1.27 (−2.19 to −0.35)). **Conclusions:** In conclusion, greater weight loss was associated with improved relative muscle strength, while adherence to a traditional diet was linked to weaker HGS. These findings highlight the importance of diet quality in maintaining muscle function after bariatric surgery.

## 1. Introduction

Obesity is a global public health crisis affecting both developed and developing countries [1]. Rapid socioeconomic development and significant lifestyle changes in recent decades have contributed to Qatar having one of the highest prevalences of overweight individuals and obesity worldwide (~70%) [1]. For individuals with severe obesity, bariatric surgery is an effective and durable treatment that induces substantial weight loss and improves or resolves many associated comorbidities [2]. In Qatar, the rising prevalence of obesity has led to an increase in bariatric surgeries; a recent report from the Qatar Biobank found that 12% of its participants had a history of bariatric surgery [3].

Despite the benefits of bariatric surgery, there are concerns about its potential impact on body composition and muscle function. The subsequent weight loss typically involves a reduction in body fat mass and lean mass, including skeletal muscle [4,5]. Skeletal muscle plays a critical role not only in overall strength and mobility but also in metabolic regulation, glucose homeostasis, and resting energy expenditure [6]. Consequently, the loss of muscle mass and function may have long-term adverse effects on health outcomes, physical independence, and quality of life [7].

Handgrip strength (HGS) is a non-invasive and practical measure of overall muscle strength and function [8]. It is widely used in clinical research [9]. Both absolute and relative HGS (RHGS) (i.e., HGS adjusted for body size, namely body weight, height or BMI) provide complementary information regarding muscle functional capacity [8]. Absolute HGS reflects total force-generating capacity, which is largely determined by skeletal muscle mass. In contrast, RHGS serves as a surrogate marker of neuromuscular efficiency or relative muscle quality. It represents the amount of strength generated per unit of body mass, which is particularly relevant for physical functions such as rising from a chair, climbing stairs, or maintaining balance [8]. Findings from epidemiological studies suggest that lower HGS predicts adverse health outcomes, such as cardiovascular disease, diabetes, sarcopenia, fragility fracture, and all-cause mortality [7]. Understanding changes in muscle strength is particularly relevant for individuals with a history of bariatric surgery, given the profound alterations in their body weight and composition. After bariatric surgery, although absolute muscle strength has been shown to decrease, relative muscle strength (adjusted for BMI and appendicular lean mass) may increase [10].

The exact mechanisms of the loss of muscle mass with age or after bariatric surgery remain to be fully established. Dietary intake is an important determinant of muscle strength in the general population [11,12,13]. At the individual nutrient and food level, specific dietary components have been linked to muscle health. For instance, high intake of salt [14] and saturated fatty acids [15] are associated with reduced muscle strength, whereas total carotenoid [16] and protein [17] are positively associated with HGS. Adherence to a balanced dietary pattern, rich in proteins, fruits, and vegetables, has been shown to be positively associated with muscle strength in the general population, whereas diets high in refined carbohydrates are negatively associated with muscle strength [11,12,13,18,19,20]. Beyond weight reduction, bariatric surgery leads to considerable changes in dietary patterns, including alterations in taste perception and food preferences [21,22]. Post-surgery, patients follow a multiphase diet emphasizing high-protein, low-calorie meals to optimize weight loss and preserve lean body mass [23,24].

A limited number of studies have examined the association between individual nutrient intake and muscle strength after bariatric surgery [25]. A meta-analysis of ten randomized clinical trials found that protein supplementation after bariatric surgery did not significantly affect BMI and lean body mass [23]. In Qatar, three distinct post-bariatric dietary patterns were identified, and a traditional dietary pattern was inversely associated with diabetes [26]. However, no studies have been conducted to assess dietary patterns’ influence on muscle strength in the post-bariatric population.

Previous post-bariatric research has largely focused on weight loss trajectories [2]; changes in body composition [4,5]; or the effects of single nutrients, such as protein, on muscle mass [17,23]. There is a limited study integrating overall dietary behavior using data-driven dietary pattern approaches derived from comprehensive food frequency questionnaires [26]. This single-nutrient or food-group approach fails to address the potential synergistic effects of habitual diet. Furthermore, functional muscle outcomes such as both absolute and relative handgrip strength have not been consistently examined in relation to broader dietary patterns and weight loss in the post-bariatric surgery population.

Therefore, we aimed to examine the association between weight loss, dietary patterns, and handgrip strength (HGS) among adults with a history of bariatric surgery using data from the Qatar Biobank study (QBB).

## 2. Materials and Methods

### 2.1. Study Sample

**Figure 1:**
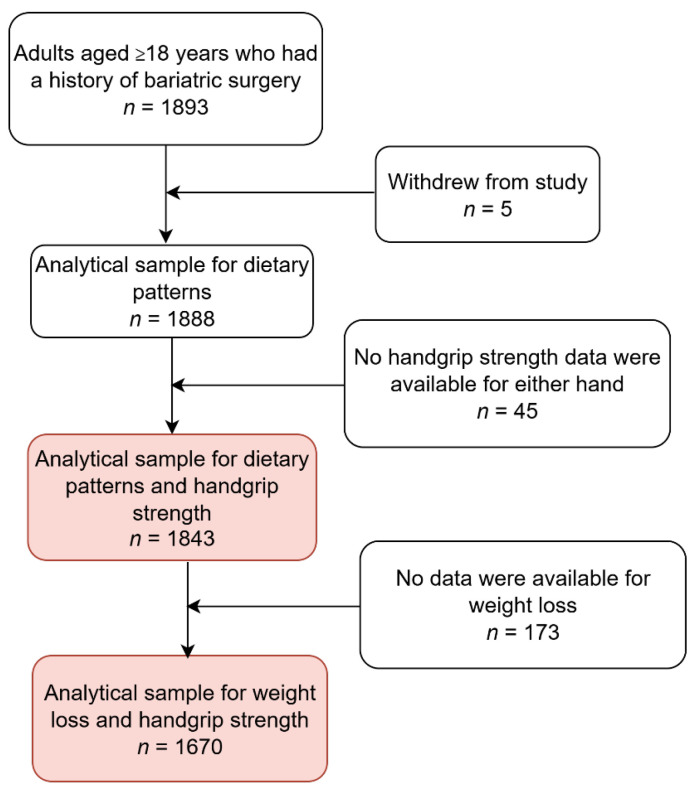
Sample flowchart of participants.

The QBB study is a large-scale, ongoing population-based study aiming to recruit 60,000 participants [27]. It enrolls Qatari nationals and long-term residents who have lived in Qatar for at least 15 years and are aged ≥18 years. By 2023, more than 37,000 adults had participated in QBB [3]. A total of 1888 participants from the Qatar Biobank (708 men and 1180 women) were included in this study (Figure 1). Eligible participants were aged ≥18 years and had a self-reported history of bariatric surgery. Three analytical samples were used: (1) dietary pattern analysis (*n* = 1888); (2) dietary patterns and handgrip strength (1843); and (3) weight loss and handgrip strength (*n* = 1670). The Qatar Biobank study received initial ethical approval from the Hamad Medical Corporation Ethics Committee in 2011, with continued approval from the Qatar Biobank Institutional Review Board (IRB) in 2017. All participants provided written informed consent prior to participation. The current study was approved under the IRB exempted category (Ex-2025-QPHI-RES-ACC-00361-0342).

### 2.2. Outcome Variables: Handgrip Strength

Handgrip strength was measured using a calibrated dynamometer during the QBB clinical assessment. Measurements were obtained with participants seated and the elbow flexed at 90 degrees, following standardized QBB protocols, with both hands assessed. Absolute HGS was calculated as the average of measurements from both hands (kg) and categorized into quartiles. Relative handgrip strength (RHGS) was calculated as absolute HGS divided by BMI. RHGS reflects muscle strength to body size. It is meaningful in the post-bariatric surgery context, where substantial weight loss occurs. Compared with absolute strength, RGHS may better capture functional muscle efficiency and performance during rapid weight change [8,10].

### 2.3. Independent Variables: Dietary Patterns and Weight Loss

A food frequency questionnaire (FFQ) was used to assess participants’ dietary patterns. The questionnaire included 102 food items adapted from the European Prospective Investigation into Cancer and Nutrition (EPIC) study. In the current analysis, the food items were grouped into 38 food groups based on similarities in nutrient composition and cooking methods. The FFQ collected information on habitual dietary intake, including the frequency of consumption of various foods and beverages and any dietary changes over the past year. The weekly consumption frequency (times per week) of the 38 food groups was used in factor analysis to identify dietary patterns. The Stata syntax for factor analysis was as follows: *factor food1-food38*, *factor(3) pcf*. The number of factors retained was determined based on eigenvalues > 1, inspection of the scree plot, and interpretability within the context of Qatari dietary habits. We attempted to extract 2 to 5 factors and decided on a three-factor solution. Varimax rotation was applied to improve interpretability (Stata syntax: *rotate*, *varimax normalize*). Factor scores for each dietary pattern were calculated for each participant by multiplying the factor loading by the standardized weekly intake frequency for each food group within that pattern and then summing these values (Stata syntax: *predict factor1 factor2 factor3*). A high factor score for a dietary pattern indicates a high intake of that pattern. Each dietary pattern score was categorized into quartiles, with quartile 1 representing the lowest intake.

Weight loss was assessed by comparing the current weight with the pre-bariatric surgery weight. Percentage of weight loss was calculated accordingly. Absolute weight loss was categorized into quartiles for analysis.

### 2.4. Covariates

The covariates included age, gender, education level (low, medium, and high), smoking status, sleep duration, snoring, leisure-time physical activity (MET hours/week, categorized into tertiles), and current dieting status. Body weight and height were measured by trained nurses, and BMI was calculated as weight (kg) divided by height squared (m^2^). The history of diabetes was self-reported, and glycated hemoglobin (HbA1C) was measured clinically. Medication use was self-reported during the nurse-administered questionnaire. The time since bariatric surgery was calculated based on the reported year of surgery. The location of bariatric surgery was categorized as within Qatar or outside Qatar. Depression syndromes were defined as a Patient Health Questionnaire (PHQ-9) score ≥ 10. Diabetes was defined as fasting blood glucose ≥ 7 mmol/L or random blood glucose ≥ 11.0 mmol/L, or HbA1c ≥ 6.5%, or self-reported diabetes [28].

### 2.5. Statistical Analysis

Descriptive statistics were used to summarize participant characteristics across quartiles of dietary pattern scores. Continuous variables were presented as means and standard deviations (SDs), and categorical variables as frequencies and percentages. Differences across quartiles were assessed using one-way analysis of variance (ANOVA) for continuous variables and chi-square tests for categorical variables. Linear regression models were used to examine associations between dietary patterns and handgrip strength (absolute and relative), as well as between weight loss quartiles and handgrip strength. Model 1 was adjusted for age and gender; Model 2 was further adjusted for education, smoking status, leisure-time physical activity, and diabetes; and Model 3 was additionally adjusted for quartiles of weight loss or dietary patterns, as appropriate. Tests for linear trends were conducted by modeling dietary pattern scores or weight loss quartiles as continuous variables.

Subgroup analyses were performed to assess potential effect modifications by sex, age group, smoking status, physical activity level, education, depression symptoms, hypertension, dieting status, and diabetes. Due to the number of missing values being small, we did not conduct multiple imputations for the missing values. All analyses were conducted using STATA 17 (Stata Corporation, College Station, TX, USA) and R version 4.5 (R Foundation for Statistical Computing, Vienna, Austria). A two-sided *p*-value < 0.05 was considered statistically significant.

## 3. Results

### 3.1. Sample Characteristics

This study included 1888 adult participants with a history of bariatric surgery, comprising 708 men (37.5%) and 1180 women (62.5%) (Table 1). Among the participants, the majority reported undergoing sleeve gastrectomy (77.3%). The mean age was 38.8 years (SD: 10.6). The mean time since bariatric surgery was 3.6 (SD 2.8) years. Nearly half of the participants (49.2%) had high educational attainment. The mean BMI was 31.6 kg/m^2^ (SD: 6.5). The average weight loss after surgery was 27.6 kg (SD: 18.0), corresponding to a 23.4% reduction (SD: 13.1. The mean absolute and relative HGS were 30.1 kg (SD: 11.2) and 1.01 (SD 0.43) (kg/BMI).

Three dietary patterns were identified using factor analysis, and their factor loadings are presented in Table S1. The Traditional pattern was characterized by higher consumption of traditional mixed dishes (such as biryani and chicken/meat/fish combinations), croissants, zaatar fatayer, lasagna, white rice, Arabic/Iranian bread, white bread, other bread, soups, potatoes, and chicken. The Prudent pattern was marked by higher intakes of salad and cooked vegetables, fresh and dried fruits, fish and seafood, fresh juices, and whole grain or brown bread. The Sweet/Fast food pattern had high intakes of desserts, ice cream, chocolate, fast food, soft drinks and nuts. The three dietary patterns explained 37.0% of the variance in food frequency intake.

Table 1 shows the sample characteristics by quartiles of the traditional dietary pattern. Participants with higher adherence to the traditional dietary pattern were younger and more likely to be female, had a medium level of education, and were more likely to sleep <5 h. There was no difference in time since bariatric surgery by levels of the traditional dietary pattern. However, both absolute and relative HGS decreased with increasing adherence to the traditional dietary pattern.

### 3.2. Association Between Different Dietary Patterns and Handgrip Strength

Higher adherence to the traditional dietary pattern was significantly associated with lower absolute HGS across all models (Table 2). In the fully adjusted model (Model 3), across the quartiles of the traditional pattern, the β coefficients (95%Cis) for absolute HGS were: 0 (reference), −0.25 (−1.15 to 0.66), −0.54 (−1.45 to 0.37), and −1.27 (−2.19 to −0.35), respectively (*p* for trend = 0.006).

Higher adherence to the prudent dietary pattern was associated with greater absolute HGS. In Model 2, adjusted for sociodemographic and lifestyle factors, compared to the lowest quartile, participants in the highest quartile of prudent dietary patterns had higher HGS (β = 1.07 kg; 95%CI: 0.18 to 1.96). In Model 3, with an additional adjustment for quartiles of weight loss, the association remained similar (β = 1.07; 95%CI 0.14 to 2.01). No significant association was found between sweet/fast food patterns and HGS.

Adherence to the traditional pattern was inversely associated with RHGS in all multivariable models (Table 3). No significant associations were found for the prudent or sweet/fast food patterns.

### 3.3. Association Between Weight Loss and Handgrip Strength

Weight loss was inversely associated with absolute HGS (Table 4). Compared with the lowest quartile, participants in the highest quartile of weight loss had 1.29 kg of lower absolute HGS (95%CI −2.26 to −0.33; *p* for trend = 0.004) after full adjustment. However, RHGS increased progressively across quartiles of weight loss (*p* for trend < 0.001), with the highest quartile showing a 0.10 kg/BMI higher value (95%CI: 0.06 to 0.13) in the fully adjusted model.

### 3.4. Subgroup Analyses

The associations between traditional and prudent patterns with HGS were consistent in subgroup analyses (Tables S2–S5). No significant interactions were found, except for a significant interaction between the prudent pattern and age in relation to absolute HGS. The positive association between the prudent pattern and HGS was mainly observed in those aged <40 years.

The inverse association between weight loss and absolute HGS was consistent across subgroups. No significant interaction was found (Table S6).

Significant interactions were found between weight loss and sex, age and diabetes in relation to RHGS (Table S7). The positive association was stronger in men, those aged < 40 years, and those without diabetes.

### 3.5. Association Between Sociodemographic and Lifestyle Factors, and Handgrip Strength

**Figure 2:**
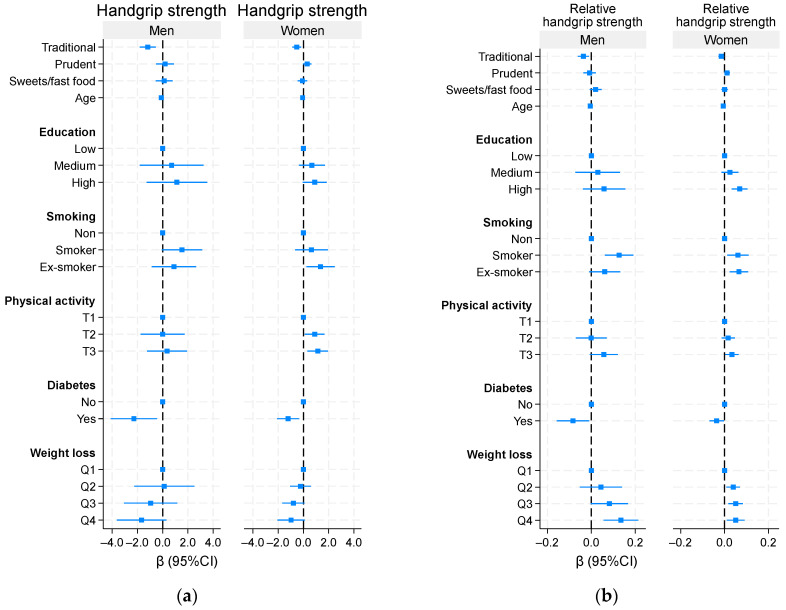
Association between sociodemographic and lifestyle factors with absolute and relative handgrip strength: (**a**) absolute handgrip strength; (**b**) relative handgrip strength. All variables in the figure were included in multivariable models.

Higher education was positively associated with HGS in women but not in men (Figure 2). Diabetes was inversely associated with RHGS in both men and women. Physical activity was positively associated with RHGS in both sexes. Smoking was positively associated with RHGS in women.

## 4. Discussion

In this cross-sectional study of 1888 adults with a history of bariatric surgery, we identified distinct and clinically meaningful associations between dietary patterns, weight loss, and handgrip strength. While substantial weight loss (mean 23.4%) was achieved, it was accompanied by lower absolute handgrip strength but higher relative handgrip strength, highlighting a divergence between muscle quantity and functional efficiency. Importantly, dietary patterns showed independent and directionally consistent associations with muscle strength: adherence to a traditional dietary pattern was associated with poorer muscle strength, whereas a prudent dietary pattern was associated with better muscle strength, particularly among younger individuals.

### 4.1. Comparison with Other Studies

#### 4.1.1. Weight Loss Post-Bariatric Surgery and Handgrip Strength

The 23.4% weight loss observed in our study is comparable to findings from other studies. For example, a U.S. study found that patients undergoing Roux-en-Y gastric bypass (RYGB) had a mean weight loss of 27.5% at 4 years after surgery [29]. Weight loss in our study was inversely associated with absolute handgrip strength but positively associated with relative handgrip strength. This pattern aligns with growing evidence that rapid post-surgical weight reduction leads to loss of lean mass and reduced absolute strength. Similar findings have been reported in previous studies where absolute strength declined approximately 2–4 kg over 6–12 months post-surgery, largely corresponding to reductions in lean soft tissue mass [10,30,31]. Studies comparing postoperative and non-surgical individuals similarly show lower absolute strength among surgical patients due to their reduced total and lean mass [31]. Prospective data further indicate that most strength loss occurs within the first 6 months, when weight loss is most rapid [10].

Although a meta-analysis found no significant association between weight loss and absolute handgrip strength (−0.46 kg; −1.76 to 0.84 kg) [32], this may reflect improvements in muscle quality despite reductions in muscle mass [4,32].

Despite weight loss being associated with decreased absolute handgrip strength in our study, the proportional improvement in strength relative to body size may reflect enhanced functional capacity. When strength was normalized to body weight, relative handgrip strength improved, reflecting better muscle efficiency and functional performance despite a lower total mass. Similar studies are consistent with our findings regarding relative handgrip strength post-bariatric surgery [4,10].

Collectively, these findings underscore the need for interventions that support lean mass preservation—such as resistance training and adequate protein intake—while reinforcing handgrip strength as a practical and informative marker of functional and metabolic recovery both before and after bariatric surgery [33].

#### 4.1.2. Dietary Patterns and Handgrip Strength

To the best of our knowledge, no study has examined the association between dietary patterns using factor analysis and handgrip strength after bariatric surgery. However, the available research suggests a complex and indirect relationship between dietary patterns and handgrip strength after bariatric surgery, with protein intake potentially playing a crucial role [25]. A recent systematic review also reported mixed and inconclusive long-term dietary effects after bariatric surgery [34].

Our findings are consistent with those from general populations. Higher adherence to a prudent dietary pattern—rich in vegetables, fruits, fish, and whole grains—was positively associated with absolute handgrip strength, consistent with evidence from multi-ethnic cohorts and studies of Mediterranean, DASH, and other high-quality dietary patterns [11,19,35,36,37,38,39].

In contrast, higher adherence to the traditional dietary pattern in our study, rich in mixed dishes, meats, and refined grains, was associated with lower absolute and relative handgrip strength. This pattern mirrors findings from Chinese, European, and UK cohorts, where diets high in red or processed meats or animal-based mixed dishes have been associated with poorer muscle strength and physical performance [11,12,40,41]. For example, a cross-sectional study in the Chinese population using data from the Tianjin Chronic Low-Grade Systemic Inflammation and Health study found that higher adherence to the animal food pattern, rich in meats and other animal-derived foods, was associated with slightly lower handgrip strength [12]. Similar inverse associations have been observed in older adults in the Newcastle 85+ Study, where a high red meat dietary pattern was associated with approximately 1.7 kg of lower grip strength [40].

The sweet/fast food pattern showed no clear association with handgrip strength after bariatric surgery. Several studies from the general population included similar patterns of Westernized, sweets, and fast food types of relations with handgrip strength [12,19,41,42,43] and are in line with our findings. A study from the INSPIRE-T cohort found that higher adherence to the “Sugar and fast food’’ dietary pattern (rich in ultra-processed, sugary, and fast foods) was common among younger adults and was linked to poorer physical performance (slower gait speed in men, and poorer chair-rise performance in women) and higher fat mass [42]. However, no consistent or statistically significant associations with handgrip strength were observed across age or sex groups [42]. Similarly, in the Hordaland Health study, neither the Western pattern (high in processed meats, sweets, and refined grains) nor the sweet pattern (high in sugary foods and drinks) showed significant associations with handgrip strength in men or women [44].

### 4.2. Potential Mechanisms

Consistent with our findings, post-bariatric surgery weight loss was associated with a decrease in handgrip strength. This decline is likely due to loss of muscle mass alongside fat mass following caloric restriction after surgery, which directly lowers the overall force-generating capacity of the hand muscles [45]. Although body weight decreases much more sharply than absolute HGS, the concurrent and more substantial reduction in body weight results in a significantly higher HGS-to-body-weight ratio [10]. This means the muscle strength remaining is more efficient relative to the patient’s new, smaller body size, leading to improved functional mobility [10,45]. To mitigate these postoperative declines in lean mass and strength, adequate protein supplementation and resistance training are recommended [46,47]. Prudent, nutrient-dense diets likely enhance handgrip strength by providing sufficient high-quality protein (including leucine) and distributed intake that supports muscle protein synthesis and preserves lean mass.

However, post-bariatric surgery physiology increases the risk of deficiencies (protein, iron, vitamin B12, fat-soluble vitamins, and others) through reduced intake and procedure-specific malabsorption. These deficiencies can impair neuromuscular function and muscle quality [48]. Furthermore, rapid and substantial weight loss can precipitate a negative nitrogen balance and downregulate muscle protein synthesis, shifting the body composition toward lean mass loss and attenuating strength, even when diet quality improves [47].

Beyond protein adequacy, high-quality dietary patterns, such as Mediterranean-like or generally nutrient-dense diets, may further enhance postoperative handgrip strength by supplying a wide matrix of “myoprotective” nutrients that support muscle fiber integrity and neuromuscular function [39]. Healthier dietary patterns provide higher levels of antioxidant vitamins, minerals, and phytochemicals that help restore redox balance and reduce oxidative stress, which is related to myofiber damage, thereby maintaining muscle quality [39].

Moreover, nutrient-dense diets offer a broader spectrum of micronutrients critical for muscle function, including magnesium, vitamin E, B-vitamins, and omega-3 fatty acids. The levels of these nutrients have been shown to differ substantially between individuals with and without sarcopenia, independent of energy intake, underscoring the critical role of diet quality in preserving muscle strength [39].

Beyond the effects of nutrient intake and lean mass loss, bariatric surgery induces broader metabolic and functional adaptations that may independently influence muscle strength. These changes differ from those observed with dietary weight loss alone and should be considered when interpreting the findings. First, procedures such as Roux-en-Y gastric bypass and sleeve gastrectomy alter gut hormones, including GLP-1, PYY, and ghrelin, which affect appetite, energy expenditure, substrate utilization, and skeletal muscle metabolism [49]. Second, rapid postoperative weight loss leads to metabolic adaptation, including a disproportionate reduction in resting energy expenditure, which may influence muscle protein turnover and contribute to lean mass loss [50]. Third, reductions in inflammatory markers such as CRP, IL-6, and TNF-α may support muscle protein synthesis [51,52]. Fourth, changes in the gut microbiome may affect muscle function through microbial metabolites and branched-chain amino acid pathways [53,54]. Overall, these adaptations suggest that diet–muscle associations occur within a markedly altered physiological context, where dietary patterns may modify or amplify postoperative metabolic changes.

Dietary patterns may reflect a broader lifestyle and health behaviors rather than acting as direct determinants of muscle strength. For example, individuals who adhere more closely to a prudent dietary pattern may also engage in more regular physical activity, have higher health literacy, or possess greater socioeconomic resources—all of which could independently influence muscle strength. Similarly, adherence to a traditional dietary pattern might cluster with other health-related behaviors or social factors that adversely affect muscle function.

### 4.3. Strengths and Limitations

This study has several strengths. First, it is the largest cohort to date (*n* = 1888) examining dietary patterns and handgrip strength among adults with a history of bariatric surgery, leveraging standardized data from the Qatar Biobank. Second, the use of factor analysis allowed for the identification of culturally relevant dietary patterns, enhancing the contextual validity of findings. Third, handgrip strength was measured using standardized dynamometry protocols, ensuring reliable assessment of muscle function. Finally, the study accounted for multiple sociodemographic and lifestyle covariates, including smoking, physical activity, and diabetes, thereby reducing residual confounding.

However, limitations should be acknowledged. The cross-sectional design precludes causal inference, and reverse causality cannot be excluded. Dietary intake was assessed using a self-reported FFQ without portion size, which may be subject to recall bias and misclassification. Although we adjusted for several covariates, including physical activity, education, and smoking, residual confounding by unmeasured or imperfectly measured lifestyle factors cannot be ruled out. Therefore, the associations reported here should be interpreted as reflecting a constellation of dietary and lifestyle behaviors rather than isolated causal effects of specific foods or nutrients. Additionally, handgrip strength was measured only once, without follow-up, and muscle mass was not directly assessed, restricting mechanistic interpretation. Finally, generalizability may be limited to Middle Eastern populations with similar dietary and cultural practices.

## 5. Conclusions

In this large cohort of Qatari adults with a history of bariatric surgery, we found that greater weight loss was associated with lower absolute but higher relative handgrip strength, reflecting improved muscle quality relative to body mass. Among dietary patterns, the traditional diet was consistently linked to weaker muscle strength, whereas the prudent diet showed a positive association that was independent of weight loss. These findings highlight the importance of diet quality, protein adequacy, and lifestyle factors in preserving muscle function after bariatric surgery. Clinically, they underscore the need for nutritional counselling and targeted interventions to optimize postoperative muscle health.

## Figures and Tables

**Table 1:** Sample characteristics by quartiles of traditional dietary pattern among adults with a history of bariatric surgery who attended the Qatar Biobank study (*n* = 1888).

	Quartiles of Traditional Dietary Pattern					
	Q1	Q2	Q3	Q4	Total	*p*-Value
N	472 (25.0%)	472 (25.0%)	472 (25.0%)	472 (25.0%)	1888 (100.0%)	
Age	40.1 (11.0)	39.7 (10.5)	38.5 (10.5)	36.7 (9.9)	38.8 (10.6)	<0.001
Gender						
Male	198 (41.9%)	167 (35.4%)	177 (37.5%)	166 (35.2%)	708 (37.5%)	0.112
Female	274 (58.1%)	305 (64.6%)	295 (62.5%)	306 (64.8%)	1180 (62.5%)	
Education						
Low	75 (15.9%)	84 (17.8%)	71 (15.0%)	70 (14.8%)	300 (15.9%)	0.042
Medium	163 (34.5%)	138 (29.2%)	169 (35.8%)	190 (40.3%)	660 (35.0%)	
High	234 (49.6%)	250 (53.0%)	232 (49.2%)	212 (44.9%)	928 (49.2%)	
Smoking						
Non	300 (63.6%)	314 (66.5%)	284 (60.2%)	298 (63.1%)	1196 (63.3%)	0.353
Smoker	96 (20.3%)	91 (19.3%)	116 (24.6%)	94 (19.9%)	397 (21.0%)	
Ex-smoker	76 (16.1%)	67 (14.2%)	72 (15.3%)	80 (16.9%)	295 (15.6%)	
Sleep duration						
<5 h	66 (14.0%)	63 (13.3%)	57 (12.1%)	88 (18.6%)	274 (14.5%)	0.049
5–7 h	238 (50.4%)	257 (54.4%)	247 (52.3%)	232 (49.2%)	974 (51.6%)	
7–8 h	130 (27.5%)	104 (22.0%)	124 (26.3%)	101 (21.4%)	459 (24.3%)	
≥8 h	38 (8.1%)	48 (10.2%)	44 (9.3%)	51 (10.8%)	181 (9.6%)	
Snore	160 (33.9%)	144 (30.5%)	161 (34.1%)	159 (33.7%)	624 (33.1%)	0.602
Leisure-time physical activity (MET hours/week)	23.2 (38.4)	19.1 (32.7)	24.2 (54.0)	25.2 (61.9)	22.9 (48.2)	0.228
BMI (kg/m^2^)	31.8 (6.8)	31.7 (6.2)	31.4 (6.5)	31.5 (6.5)	31.6 (6.5)	0.785
BMI categories						
Normal	65 (13.8%)	55 (11.7%)	74 (15.7%)	57 (12.1%)	251 (13.3%)	0.286
Overweight	135 (28.6%)	154 (32.6%)	149 (31.6%)	162 (34.3%)	600 (31.8%)	
Obese	272 (57.6%)	263 (55.7%)	249 (52.8%)	253 (53.6%)	1037 (54.9%)	
Traditional pattern	−0.9 (0.3)	−0.4 (0.1)	0.0 (0.2)	1.3 (1.0)	0.0 (1.0)	<0.001
Prudent pattern	0.2 (1.0)	−0.1 (0.8)	−0.1 (0.8)	0.1 (1.3)	0.0 (1.0)	<0.001
Sweet/fast food pattern	0.1 (1.0)	−0.1 (0.8)	−0.1 (0.8)	0.1 (1.3)	−0.0 (1.0)	<0.001
On diet	147 (31.1%)	111 (23.5%)	100 (21.2%)	110 (23.3%)	468 (24.8%)	0.002
Fruit intake (times/week)	9.1 (8.3)	6.5 (6.6)	7.2 (7.1)	9.4 (8.8)	8.0 (7.8)	<0.001
Vegetable intake (times/week)	17.1 (15.1)	15.4 (13.8)	18.4 (15.5)	29.4 (24.5)	20.1 (18.6)	<0.001
Soft drink (times/week)	3.4 (6.5)	3.2 (5.9)	3.9 (6.0)	6.3 (9.3)	4.2 (7.2)	<0.001
HbA1C (mmol/L)	5.6 (1.0)	5.5 (1.0)	5.5 (0.9)	5.4 (0.8)	5.5 (0.9)	0.054
HbA1C ≥ 7 (mmol/L)	34 (7.3%)	31 (6.6%)	27 (5.8%)	22 (4.7%)	114 (6.1%)	0.396
Hypertension	84 (17.8%)	60 (12.7%)	49 (10.4%)	48 (10.2%)	241 (12.8%)	0.001
Self-reported history of diabetes	94 (19.9%)	93 (19.7%)	82 (17.4%)	79 (16.7%)	348 (18.4%)	0.484
Diabetes	100 (21.3%)	96 (20.4%)	86 (18.4%)	80 (17.1%)	362 (19.3%)	0.347
Depression symptom	94 (19.9%)	97 (20.6%)	121 (25.6%)	164 (34.7%)	476 (25.2%)	<0.001
Diabetes medication other than insulin	49 (10.4%)	49 (10.4%)	41 (8.7%)	31 (6.6%)	170 (9.0%)	0.129
Insulin use	23 (4.9%)	21 (4.4%)	17 (3.6%)	14 (3.0%)	75 (4.0%)	0.439
Hypertension medication use	58 (12.3%)	47 (10.0%)	39 (8.3%)	34 (7.2%)	178 (9.4%)	0.043
Weight loss (kg)	27.2 (18.5)	27.4 (17.2)	28.0 (18.3)	27.6 (18.1)	27.6 (18.0)	0.934
Percentage of weight loss	22.8 (13.5)	23.5 (12.6)	23.7 (13.2)	23.7 (13.3)	23.4 (13.1)	0.716
Handgrip strength (kg)	31.5 (11.8)	30.0 (11.3)	30.2 (11.3)	28.8 (10.5)	30.1 (11.2)	0.004
Relative handgrip strength (kg/BMI)	1.04 (0.45)	0.99 (0.43)	1.01 (0.45)	0.95 (0.39)	1.00 (0.43)	0.012
Location of bariatric surgery						
Qatar	258 (55.2%)	279 (59.5%)	263 (56.7%)	274 (59.4%)	1074 (57.7%)	0.469
Outside Qatar	209 (44.8%)	190 (40.5%)	201 (43.3%)	187 (40.6%)	787 (42.3%)	
Time since bariatric surgery (years)	3.4 (3.1)	3.6 (2.6)	3.7 (2.8)	3.7 (2.7)	3.6 (2.8)	0.370

Values are means (SD), or *n* (%). *p*-values were based on χ^2^ test for categorized variables and ANOVA for continuous variables.

**Table 2:** Association between dietary patterns and absolute handgrip strength among adults with a history of bariatric surgery who attended the Qatar Biobank study (*n* = 1848).

	Quartiles of Dietary Pattern				
	Q1	Q2	Q3	Q4	*p* for Trend *
Traditional pattern					
Model 1	0.00	−0.23 (−1.10 to 0.64)	−0.44 (−1.30 to 0.43)	−1.55 (−2.42 to −0.68)	<0.001
Model 2	0.00	−0.26 (−1.13 to 0.61)	−0.50 (−1.36 to 0.37)	−1.47 (−2.34 to −0.59)	0.001
Model 3	0.00	−0.25 (−1.15 to 0.66)	−0.54 (−1.45 to 0.37)	−1.27 (−2.19 to −0.35)	0.006
Prudent pattern					
Model 1	0.00	0.75 (−0.12 to 1.62)	0.89 (0.02 to 1.77)	1.15 (0.26 to 2.04)	0.012
Model 2	0.00	0.67 (−0.20 to 1.54)	0.85 (−0.03 to 1.72)	1.07 (0.18 to 1.96)	0.019
Model 3	0.00	0.89 (−0.01 to 1.79)	0.75 (−0.16 to 1.66)	1.07 (0.14 to 2.01)	0.041
Sweets/fast food pattern					
Model 1	0.00	0.75 (−0.12 to 1.61)	1.32 (0.44 to 2.20)	0.79 (−0.12 to 1.69)	0.043
Model 2	0.00	0.53 (−0.34 to 1.40)	1.20 (0.32 to 2.08)	0.59 (−0.31 to 1.50)	0.092
Model 3	0.00	0.61 (−0.31 to 1.52)	1.22 (0.30 to 2.14)	0.43 (−0.52 to 1.38)	0.212

Values were regression coefficients (95%CI) from linear regression. Model 1 was adjusted for age and sex. Model 2 was further adjusted for education, smoking, physical activity and diabetes. Model 3 was further adjusted for quartiles of weight loss. * *p* for trend was tested using dietary pattern scores as a continuous variable in the linear model.

**Table 3:** Association between dietary patterns and relative handgrip strength among adults with a history of bariatric surgery who attended the Qatar Biobank study (*n* = 1848).

	Quartiles of Dietary Pattern				
	Q1	Q2	Q3	Q4	*p* for Trend *
Traditional pattern					
Model 1	0.00	−0.01 (−0.04 to 0.03)	−0.01 (−0.04 to 0.03)	−0.07 (−0.10 to −0.03)	<0.001
Model 2	0.00	−0.01 (−0.05 to 0.02)	−0.01 (−0.05 to 0.02)	−0.06 (−0.10 to −0.03)	<0.001
Model 3	0.00	−0.01 (−0.05 to 0.03)	−0.02 (−0.05 to 0.02)	−0.05 (−0.09 to −0.01)	0.006
Prudent pattern					
Model 1	0.00	0.00 (−0.04 to 0.04)	0.01 (−0.02 to 0.05)	0.03 (−0.00 to 0.07)	0.057
Model 2	0.00	−0.00 (−0.04 to 0.03)	0.01 (−0.02 to 0.05)	0.03 (−0.01 to 0.06)	0.086
Model 3	0.00	0.01 (−0.02 to 0.05)	0.02 (−0.02 to 0.05)	0.02 (−0.01 to 0.06)	0.193
Sweet/fast food pattern					
Model 1	0.00	0.04 (0.00 to 0.08)	0.04 (0.01 to 0.08)	0.04 (0.01 to 0.08)	0.025
Model 2	0.00	0.03 (−0.01 to 0.07)	0.04 (0.00 to 0.07)	0.03 (−0.01 to 0.07)	0.091
Model 3	0.00	0.03 (−0.00 to 0.07)	0.04 (0.00 to 0.07)	0.03 (−0.00 to 0.07)	0.095

Values were regression coefficients (95%CI) from linear regression. Model 1 was adjusted for age and sex. Model 2 was further adjusted for education, smoking, physical activity and diabetes. Model 3 was further adjusted for quartiles of weight loss. * *p* for trend was tested using dietary pattern scores as a continuous variable in the linear model.

**Table 4:** Association between weight loss and handgrip strength among adults with a history of bariatric surgery who attended the Qatar Biobank study (*n* = 1670).

	Quartiles of Weight Loss				
	Q1	Q2	Q3	Q4	*p* for Trend *
Handgrip strength					
Model 1	0.00	−0.05 (−0.95 to 0.86)	−0.81 (−1.72 to 0.09)	−1.15 (−2.11 to −0.18)	0.008
Model 2	0.00	−0.12 (−1.02 to 0.78)	−0.81 (−1.72 to 0.09)	−1.26 (−2.23 to −0.29)	0.005
Model 3	0.00	−0.15 (−1.04 to 0.75)	−0.84 (−1.74 to 0.06)	−1.29 (−2.26 to −0.33)	0.004
Relative handgrip strength					
Model 1	0.00	0.05 (0.01 to 0.08)	0.06 (0.03 to 0.10)	0.11 (0.07 to 0.15)	<0.001
Model 2	0.00	0.04 (0.01 to 0.08)	0.06 (0.02 to 0.10)	0.10 (0.06 to 0.13)	<0.001
Model 3	0.00	0.04 (0.01 to 0.08)	0.06 (0.02 to 0.09)	0.10 (0.06 to 0.13)	<0.001

Values were regression coefficients (95%CI) from linear regression. Model 1 was adjusted for age and sex. Model 2 was further adjusted for education, smoking, physical activity and diabetes. Model 3 was further adjusted for quartiles of dietary patterns (continuous scores). * *p* for trend was tested using quartiles of weight loss as a continuous variable in the linear model.

## Data Availability

The data that support the findings of this study are available from the Qatar Biobank study, but restrictions apply to the availability of these data, which were used under license for the current study, and so are not publicly available. Data are however available from the authors upon reasonable request and with permission of the Qatar Biobank study.

## Supplementary Materials

The following supporting information can be downloaded at: https://www.mdpi.com/article/10.3390/nu18091411/s1, Table S1: Factor loadings of dietary patterns among adults with a history of bariatric surgery who attended Qatar Biobank study (*n* = 1888); Table S2: Subgroup analyses of the association between traditional dietary pattern and handgrip strength; Table S3: Subgroup analyses of the association between traditional dietary pattern and relative handgrip strength; Table S4: Subgroup analyses of the association between Prudent dietary pattern and handgrip strength; Table S5: Subgroup analyses of the association between Prudent dietary pattern and relative handgrip strength; Table S6: Subgroup analyses of the association between quartiles of weight loss and handgrip strength; Table S7: Subgroup analyses of the association between quartiles of weight loss and relative handgrip strength.

## Abbreviations

The following abbreviations are used in this manuscript:

HGS	Handgrip strength
RHGS	Relative handgrip strength
QBB	Qatar Biobank study
FFQ	Food frequency questionnaire

## References

[1] GBD Adult BMI Collaborators (2025). Global, regional, and national prevalence of adult overweight and obesity, 1990–2021, with forecasts to 2050: A forecasting study for the Global Burden of Disease Study 2021. Lancet.

[2] Courcoulas A.P., King W.C., Belle S.H., Berk P., Flum D.R., Garcia L., Gourash W., Horlick M., Mitchell J.E., Pomp A. (2018). Seven-Year Weight Trajectories and Health Outcomes in the Longitudinal Assessment of Bariatric Surgery (LABS) Study. JAMA Surg..

[3] Qatar Precicion Health Institute Qatar Biobank Annual Report 2022–2023. https://www.qphi.org.qa/media-centre/annual-report.

[4] Zhou N., Scoubeau C., Forton K., Loi P., Closset J., Deboeck G., Moraine J.J., Klass M., Faoro V. (2022). Lean Mass Loss and Altered Muscular Aerobic Capacity after Bariatric Surgery. Obes. Facts.

[5] Hua M., Li J., Wang T., Xu Y., Zhao Y., Sun Q., Yuan H., Wang D. (2025). Meta-analysis of changes in skeletal muscle mass within 1 year after bariatric surgery. Surg. Endosc..

[6] Jin J.B., Robinson A., Soukup T., Black E., Abit A., Hammer S.M., Han A., Lucas E., Kim Y., Bae J. (2025). Metabolic and molecular regulation in skeletal muscle dysfunction and regeneration. Front. Cell Dev. Biol..

[7] Vaishya R., Misra A., Vaish A., Ursino N., D’Ambrosi R. (2024). Hand grip strength as a proposed new vital sign of health: A narrative review of evidences. J. Health Popul. Nutr..

[8] Szaflik P., Zadoń H., Michnik R., Nowakowska-Lipiec K. (2025). Handgrip Strength as an Indicator of Overall Strength and Functional Performance—Systematic Review. Appl. Sci..

[9] Xie H., Ruan G., Deng L., Zhang H., Ge Y., Zhang Q., Lin S., Song M., Zhang X., Liu X. (2022). Comparison of absolute and relative handgrip strength to predict cancer prognosis: A prospective multicenter cohort study. Clin. Nutr..

[10] Alba D.L., Wu L., Cawthon P.M., Mulligan K., Lang T., Patel S., King N.J., Carter J.T., Rogers S.J., Posselt A.M. (2019). Changes in Lean Mass, Absolute and Relative Muscle Strength, and Physical Performance After Gastric Bypass Surgery. J. Clin. Endocrinol. Metab..

[11] Gedmantaite A., Celis-Morales C.A., Ho F., Pell J.P., Ratkevicius A., Gray S.R. (2020). Associations between diet and handgrip strength: A cross-sectional study from UK Biobank. Mech. Ageing Dev..

[12] Zhang X., Gu Y., Cheng J., Meng G., Zhang Q., Liu L., Wu H., Zhang S., Wang Y., Zhang T. (2021). The relationship between dietary patterns and grip strength in the general population: The TCLSIH cohort study. Eur. J. Nutr..

[13] Mazza E., Ferro Y., Maurotti S., Micale F., Boragina G., Russo R., Lascala L., Sciacqua A., Gazzaruso C., Montalcini T. (2024). Association of dietary patterns with sarcopenia in adults aged 50 years and older. Eur. J. Nutr..

[14] Yoshida Y., Kosaki K., Sugasawa T., Matsui M., Yoshioka M., Aoki K., Kuji T., Mizuno R., Kuro O.M., Yamagata K. (2020). High Salt Diet Impacts the Risk of Sarcopenia Associated with Reduction of Skeletal Muscle Performance in the Japanese Population. Nutrients.

[15] Arias-Fernandez L., Struijk E.A., Rodriguez-Artalejo F., Lopez-Garcia E., Lana A. (2020). Habitual dietary fat intake and risk of muscle weakness and lower-extremity functional impairment in older adults: A prospective cohort study. Clin. Nutr..

[16] Sahni S., Dufour A.B., Fielding R.A., Newman A.B., Kiel D.P., Hannan M.T., Jacques P.F. (2021). Total carotenoid intake is associated with reduced loss of grip strength and gait speed over time in adults: The Framingham Offspring Study. Am. J. Clin. Nutr..

[17] Houston D.K., Nicklas B.J., Ding J., Harris T.B., Tylavsky F.A., Newman A.B., Lee J.S., Sahyoun N.R., Visser M., Kritchevsky S.B. (2008). Dietary protein intake is associated with lean mass change in older, community-dwelling adults: The Health, Aging, and Body Composition (Health ABC) Study. Am. J. Clin. Nutr..

[18] Baik I. (2025). Associations of dietary and physical activity behaviors with handgrip strength in middle-aged and older adults: A prospective cohort study. Sci. Rep..

[19] Lee S. (2020). Associations Between Dietary Patterns and Handgrip Strength: The Korea National Health and Nutrition Examination Survey 2014-2016. J. Am. Coll. Nutr..

[20] Ma Z., Yang H., Meng G., Zhang Q., Liu L., Wu H., Gu Y., Zhang S., Wang X., Zhang J. (2023). Anti-inflammatory dietary pattern is associated with handgrip strength decline: A prospective cohort study. Eur. J. Nutr..

[21] Coluzzi I., Raparelli L., Guarnacci L., Paone E., Del Genio G., le Roux C.W., Silecchia G. (2016). Food Intake and Changes in Eating Behavior After Laparoscopic Sleeve Gastrectomy. Obes. Surg..

[22] Althumiri N.A., Basyouni M.H., Al-Qahtani F.S., Zamakhshary M., BinDhim N.F. (2021). Food taste, dietary consumption, and food preference perception of changes following bariatric surgery in the Saudi population: A cross-sectional study. Nutrients.

[23] Ranjbar M., Fallah M., Djafarian K., Mohammadi H., Mohammadi Farsani G., Shab-Bidar S. (2025). The effects of protein supplementation on body composition after bariatric surgery: A systematic review and meta-analysis of randomized controlled trials. Obesity.

[24] Parrott J., Frank L., Rabena R., Craggs-Dino L., Isom K.A., Greiman L. (2017). American Society for Metabolic and Bariatric Surgery Integrated Health Nutritional Guidelines for the Surgical Weight Loss Patient 2016 Update: Micronutrients. Surg. Obes. Relat. Dis..

[25] Smelt H.J.M., Pouwels S., Celik A., Gupta A., Smulders J.F. (2019). Assessment of Physical Fitness after Bariatric Surgery and Its Association with Protein Intake and Type of Cholecalciferol Supplementation. Medicina.

[26] Almaghrbi R., Alyamani R., Aliwi L., Moawad J., Hussain A., Wang Y., Shi Z. (2024). Association between Dietary Pattern, Weight Loss, and Diabetes among Adults with a History of Bariatric Surgery: Results from the Qatar Biobank Study. Nutrients.

[27] Al Thani A., Fthenou E., Paparrodopoulos S., Al Marri A., Shi Z., Qafoud F., Afifi N. (2019). Qatar Biobank Cohort Study: Study Design and First Results. Am. J. Epidemiol..

[28] American Diabetes A. (2014). Diagnosis and classification of diabetes mellitus. Diabetes Care.

[29] Maciejewski M.L., Arterburn D.E., Van Scoyoc L., Smith V.A., Yancy W.S., Weidenbacher H.J., Livingston E.H., Olsen M.K. (2016). Bariatric surgery and long-term durability of weight loss. JAMA Surg..

[30] Ibacache-Saavedra P., Martínez-Rosales E., Jerez-Mayorga D., Miranda-Fuentes C., Artero E.G., Cano-Cappellacci M. (2024). Effects of bariatric surgery on muscle strength and quality: A systematic review and meta-analysis. Obes. Rev..

[31] de Oliveira P.A.P., Montenegro A.C.P., Bezerra L.R.A., da Conceição Chaves de Lemos M., Bandeira F. (2020). Body composition, serum sclerostin and physical function after bariatric surgery: Performance of dual-energy X-ray absorptiometry and multifrequency bioelectrical impedance analysis. Obes. Surg..

[32] Jung H.N., Kim S.-O., Jung C.H., Lee W.J., Kim M.J., Cho Y.K. (2023). Preserved muscle strength despite muscle mass loss after bariatric metabolic surgery: A systematic review and meta-analysis. Obes. Surg..

[33] Otto M., Kautt S., Kremer M., Kienle P., Post S., Hasenberg T. (2014). Handgrip strength as a predictor for post bariatric body composition. Obes. Surg..

[34] Cheung H., Strodl E., Musial J., MacLaughlin H., Byrnes A., Lewis C., Ross L. (2023). Associations between diet composition, dietary pattern, and weight outcomes after bariatric surgery: A systematic review. Int. J. Obes..

[35] Kuczmarski M.F., Orsega-Smith E., Beydoun M.A., Evans M.K., Zonderman A.B. (2025). Protein Intake and Diet Quality Mediate the Relationship Between Sleep and Handgrip Strength in Adults in the HANDLS Study. Nutrients.

[36] Huang J., Shanmugam A., Huang X., van Dam R.M., Hilal S. (2024). Association of diet quality with hand grip strength weakness and asymmetry in a multi-ethnic Asian cohort. Br. J. Nutr..

[37] Fanelli Kuczmarski M., Pohlig R.T., Stave Shupe E., Zonderman A.B., Evans M.K. (2018). Dietary Protein Intake and Overall Diet Quality Are Associated with Handgrip Strength in African American and White Adults. J. Nutr. Health Aging.

[38] Pikosky M.A., Cifelli C.J., Agarwal S., Fulgoni V.L. (2022). Association of dietary protein intake and grip strength among adults aged 19+ years: NHANES 2011–2014 analysis. Front. Nutr..

[39] Granic A., Sayer A.A., Robinson S.M. (2019). Dietary patterns, skeletal muscle health, and sarcopenia in older adults. Nutrients.

[40] Granic A., Jagger C., Davies K., Adamson A., Kirkwood T., Hill T.R., Siervo M., Mathers J.C., Sayer A.A. (2016). Effect of dietary patterns on muscle strength and physical performance in the very old: Findings from the Newcastle 85+ study. PLoS ONE.

[41] Kim I., Son K., Jeong S.J., Lim H. (2022). Sex and diet-related disparities in low handgrip strength among young and middle-aged Koreans: Findings based on the Korea National Health and Nutrition Examination Survey (KNHANES) from 2014 to 2017. Nutrients.

[42] Lengelé L., de França N.A.G., Rolland Y., Guyonnet S., de Souto Barreto P., Vellas B. (2023). Body composition, physical function, and dietary patterns in people from 20 to over 80 years old: The INSPIRE-T cohort. Res. Sq..

[43] Samadi M., Khosravy T., Azadbakht L., Rezaei M., Mosafaghadir M., Kamari N., Bagheri A., Pasdar Y., Najafi F., Hamze B. (2021). Major dietary patterns in relation to muscle strength status among middle-aged people: A cross-sectional study within the RaNCD cohort. Food Sci. Nutr..

[44] Sabir Z., Dierkes J., Hjartåker A., Rosendahl-Riise H. (2023). The association of dietary patterns with muscle mass and strength in old age: The Hordaland Health Study. Eur. J. Nutr..

[45] Gerken A., Rohr-Kräutle K.-K., Weiss C., Seyfried S., Reißfelder C., Vassilev G., Otto M. (2021). Handgrip strength and phase angle predict outcome after bariatric surgery. Obes. Surg..

[46] Lakdawala M., Fernando R., Surendran S., Jaiswal R. (2025). Protein Supplementation Post-Metabolic and Bariatric Surgery. Handbook of Bariatric Nutrition.

[47] Faria I., Samreen S., McTaggart L., Arentson-Lantz E.J., Murton A.J. (2024). The Etiology of Reduced Muscle Mass with Surgical and Pharmacological Weight Loss and the Identification of Potential Countermeasures. Nutrients.

[48] Herron D.M., Herrington H. (2025). Bariatric Surgery: Postoperative Nutritional Management. https://www.uptodate.com/contents/bariatric-surgery-postoperative-nutritional-management.

[49] le Roux C.W., Welbourn R., Werling M., Osborne A., Kokkinos A., Laurenius A., Lonroth H., Fandriks L., Ghatei M.A., Bloom S.R. (2007). Gut hormones as mediators of appetite and weight loss after Roux-en-Y gastric bypass. Ann. Surg..

[50] Knuth N.D., Johannsen D.L., Tamboli R.A., Marks-Shulman P.A., Huizenga R., Chen K.Y., Abumrad N.N., Ravussin E., Hall K.D. (2014). Metabolic adaptation following massive weight loss is related to the degree of energy imbalance and changes in circulating leptin. Obesity.

[51] Illan-Gomez F., Gonzalvez-Ortega M., Orea-Soler I., Alcaraz-Tafalla M.S., Aragon-Alonso A., Pascual-Diaz M., Perez-Paredes M., Lozano-Almela M.L. (2012). Obesity and inflammation: Change in adiponectin, C-reactive protein, tumour necrosis factor-alpha and interleukin-6 after bariatric surgery. Obes. Surg..

[52] Rao S.R. (2012). Inflammatory markers and bariatric surgery: A meta-analysis. Inflamm. Res..

[53] Liu R., Hong J., Xu X., Feng Q., Zhang D., Gu Y., Shi J., Zhao S., Liu W., Wang X. (2017). Gut microbiome and serum metabolome alterations in obesity and after weight-loss intervention. Nat. Med..

[54] Furet J.P., Kong L.C., Tap J., Poitou C., Basdevant A., Bouillot J.L., Mariat D., Corthier G., Dore J., Henegar C. (2010). Differential adaptation of human gut microbiota to bariatric surgery-induced weight loss: Links with metabolic and low-grade inflammation markers. Diabetes.

