# Supplementary material for: Association Between Dietary Patterns, Weight Loss, and Handgrip Strength Among Qatari Adults with a History of Bariatric Surgery: Results from the Qatar Biobank Study
Source: Nutrients. 2026 Apr 29;18(9):1411. doi: 10.3390/nu18091411 (PMC13165315; doi:10.3390/nu18091411)
Supplement: Supplementary file 1 [file nutrients-18-01411-s001.zip › nutrients-4257245-supplementary.pdf]

Table S1 Factor loadings of dietary patterns among adults with a history of bariatric surgery who attended Qatar Biobank Study (n=1888)

Food	Traditional pattern	Prudent pattern	Sweet/Fast food pattern
Croissant	0.66	-0.01	0.31
Zaatar fatayer	0.65	0.05	0.29
White bread	0.61	0.05	0.21
Other bread	0.60	0.04	0.18
Arabic/Iranian bread	0.59	0.16	0.19
Cheese	0.55	0.13	0.17
Yoghurt	0.52	0.41	-0.10
Brown bread	0.48	0.29	-0.05
Milk shakes	0.47	0.22	-0.02
Butter	0.45	0.05	0.19
Breakfast Cereal	0.43	0.34	0.13
Milk added to cereal	0.42	0.25	-0.05
Biryani	0.41	0.34	0.35
Coffee	0.34	0.06	0.12
Tea	0.32	0.07	0.08
Milk	0.32	0.17	0.02
Fresh fruit	0.09	0.69	0.08
Salad and raw vegetables	0.29	0.67	0.00
Grilled/fried/baked Fish	0.04	0.63	0.13
Fish	0.05	0.61	0.17
Canned/dried fruit and dates	0.11	0.60	-0.01
Fresh fruit juice	0.16	0.56	0.21
Salad and cooked vegetables	0.38	0.55	0.10

Soups/starters	0.50	0.51	0.28
Potato	0.34	0.36	0.22
Asian noodle	0.34	0.35	0.32
Eggs	0.11	0.32	0.22
White rice	0.30	0.31	0.21
Desserts	0.14	0.12	0.70
Chocolate	0.05	-0.08	0.68
Fast food	0.36	0.21	0.66
Ice cream	0.10	0.13	0.65
Soft drink	0.21	0.04	0.56
Chicken/meat fish mixed dish	0.34	0.46	0.48
Nuts	-0.03	0.33	0.47
Lasagna	0.40	0.22	0.45
Red meat	0.17	0.30	0.32
Chicken	0.18	0.26	0.30
Variance explained (%)	26.0	6.0	5.0

Table S2 Subgroup analyses of the association between traditional dietary pattern and handgrip strength

	Quartiles of Traditional					
	Q1	Q2	Q3	Q4	p for trend	p for interaction
Gender						0.290
Male	0.00	-0.69 (-2.44 to 1.06)	-0.82 (-2.54 to 0.90)	-2.80 (-4.58 to -1.03)	0.004	
Female	0.00	-0.20 (-1.08 to 0.67)	-0.49 (-1.38 to 0.39)	-1.00 (-1.88 to -0.13)	0.019	
Age ≥40 (years)						0.730
No	0.00	-0.88 (-2.07 to 0.32)	-0.69 (-1.87 to 0.48)	-1.88 (-3.03 to -0.74)	0.003	
Yes	0.00	-0.26 (-1.48 to 0.95)	-1.00 (-2.24 to 0.24)	-1.64 (-2.96 to -0.32)	0.008	
Smoking						0.253
Non	0.00	-0.36 (-1.33 to 0.60)	-0.51 (-1.50 to 0.48)	-0.90 (-1.88 to 0.08)	0.072	
Smoker	0.00	-0.86 (-3.07 to 1.34)	-1.24 (-3.31 to 0.82)	-3.46 (-5.68 to -1.24)	0.003	
Ex-smoker	0.00	0.08 (-2.51 to 2.67)	-0.95 (-3.51 to 1.62)	-2.32 (-4.84 to 0.19)	0.052	
Leisure time PA (MET hours/week)						0.547
T1 (low)	0.00	-0.47 (-1.79 to 0.86)	0.23 (-1.10 to 1.56)	-1.47 (-2.80 to -0.13)	0.084	
T2	0.00	-0.42 (-2.00 to 1.17)	-1.87 (-3.52 to -0.22)	-2.01 (-3.63 to -0.40)	0.004	
T3 (high)	0.00	-0.44 (-2.05 to 1.17)	-0.98 (-2.55 to 0.60)	-1.77 (-3.39 to -0.15)	0.026	
Education						0.655
Low	0.00	0.12 (-2.00 to 2.23)	-1.99 (-4.18 to 0.21)	-1.44 (-3.66 to 0.79)	0.067	
Medium	0.00	-0.20 (-1.75 to 1.35)	0.04 (-1.45 to 1.52)	-1.07 (-2.52 to 0.37)	0.182	
High	0.00	-0.70 (-1.89 to 0.49)	-0.76 (-1.96 to 0.45)	-2.02 (-3.27 to -0.78)	0.003	
Depression symptom						0.974
No	0.00	-0.53 (-1.51 to 0.46)	-0.76 (-1.75 to 0.24)	-1.69 (-2.73 to -0.64)	0.002	
Yes	0.00	0.13 (-1.63 to 1.90)	-0.19 (-1.88 to 1.49)	-1.27 (-2.86 to 0.32)	0.072	
Hypertension						0.956
No	0.00	-0.38 (-1.31 to 0.55)	-0.61 (-1.53 to 0.31)	-1.64 (-2.57 to -0.72)	<0.001	
Yes	0.00	-1.16 (-3.52 to 1.20)	-1.57 (-4.06 to 0.92)	-2.13 (-4.75 to 0.48)	0.081	
On diet						0.127
No	0.00	-0.49 (-1.47 to 0.49)	-0.27 (-1.25 to 0.71)	-1.29 (-2.28 to -0.31)	0.021	

Yes	0.00	0.43 (-1.37 to 2.23)	-1.59 (-3.41 to 0.24)	-2.67 (-4.48 to -0.85)	0.001	
Diabetes						0.924
No	0.00	-0.52 (-1.46 to 0.42)	-0.73 (-1.67 to 0.21)	-1.74 (-2.68 to -0.80)	<0.001	
Yes	0.00	-0.19 (-2.29 to 1.91)	-0.53 (-2.68 to 1.62)	-1.16 (-3.35 to 1.03)	0.285	

Values were regression coefficients (95%CI) from linear regression. The p values for trend were tested using quartiles of dietary patterns as ordinal variables in the models. Model adjusted for age, sex, education, smoking, physical activity and diabetes.

Table S3 Subgroup analyses of the association between traditional dietary pattern and relative handgrip strength

	Quartiles of Traditional					
	Q1	Q2	Q3	Q4	p for trend	p for interaction
Gender						0.058
Male	0.00	-0.02 (-0.10 to 0.05)	-0.02 (-0.09 to 0.05)	-0.13 (-0.20 to -0.05)	0.002	
Female	0.00	-0.01 (-0.05 to 0.02)	-0.02 (-0.05 to 0.02)	-0.03 (-0.07 to 0.00)	0.057	
Age ≥40 (years)						0.183
No	0.00	-0.04 (-0.09 to 0.01)	-0.02 (-0.07 to 0.03)	-0.09 (-0.14 to -0.04)	0.002	
Yes	0.00	0.00 (-0.05 to 0.05)	-0.03 (-0.08 to 0.01)	-0.05 (-0.10 to 0.00)	0.031	
Smoking						0.327
Non	0.00	-0.00 (-0.04 to 0.04)	-0.01 (-0.04 to 0.03)	-0.03 (-0.07 to 0.01)	0.143	
Smoker	0.00	-0.06 (-0.16 to 0.03)	-0.05 (-0.14 to 0.04)	-0.14 (-0.23 to -0.05)	0.008	
Ex-smoker	0.00	-0.02 (-0.13 to 0.08)	-0.06 (-0.17 to 0.04)	-0.15 (-0.25 to -0.05)	0.003	
Leisure time PA (MET hours/week)						0.675
T1 (low)	0.00	-0.04 (-0.09 to 0.01)	-0.00 (-0.05 to 0.05)	-0.08 (-0.13 to -0.02)	0.029	
T2	0.00	-0.00 (-0.06 to 0.06)	-0.02 (-0.09 to 0.04)	-0.06 (-0.12 to 0.01)	0.061	
T3 (high)	0.00	-0.01 (-0.07 to 0.06)	-0.05 (-0.11 to 0.02)	-0.07 (-0.14 to -0.00)	0.021	
Education						0.668
Low	0.00	-0.02 (-0.10 to 0.06)	-0.04 (-0.12 to 0.04)	-0.04 (-0.12 to 0.05)	0.311	
Medium	0.00	-0.04 (-0.11 to 0.02)	-0.01 (-0.07 to 0.05)	-0.06 (-0.12 to 0.00)	0.146	
High	0.00	-0.00 (-0.05 to 0.05)	-0.02 (-0.07 to 0.03)	-0.08 (-0.13 to -0.03)	0.002	
Depression symptom						0.261
No	0.00	-0.02 (-0.06 to 0.02)	-0.04 (-0.08 to 0.00)	-0.07 (-0.12 to -0.03)	<0.001	
Yes	0.00	-0.02 (-0.09 to 0.06)	0.04 (-0.03 to 0.11)	-0.04 (-0.10 to 0.03)	0.424	
Hypertension						0.550
No	0.00	-0.02 (-0.06 to 0.02)	-0.02 (-0.06 to 0.02)	-0.08 (-0.12 to -0.04)	<0.001	
Yes	0.00	-0.03 (-0.11 to 0.05)	-0.04 (-0.12 to 0.05)	-0.01 (-0.10 to 0.08)	0.624	
On diet						0.160
No	0.00	-0.01 (-0.05 to 0.03)	-0.00 (-0.04 to 0.04)	-0.05 (-0.09 to -0.01)	0.043	

Yes	0.00	-0.00 (-0.08 to 0.07)	-0.05 (-0.13 to 0.02)	-0.13 (-0.20 to -0.05)	<0.001	
Diabetes						0.796
No	0.00	-0.01 (-0.05 to 0.02)	-0.02 (-0.06 to 0.01)	-0.07 (-0.11 to -0.03)	<0.001	
Yes	0.00	-0.03 (-0.11 to 0.05)	0.01 (-0.07 to 0.09)	-0.04 (-0.12 to 0.04)	0.531	

Values were regression coefficients (95%CI) from linear regression. The p values for trend were tested using quartiles of dietary patterns as ordinal variables in the models. Model adjusted for age, sex, education, smoking, physical activity and diabetes.

Table S4 Subgroup analyses of the association between Prudent dietary pattern and handgrip strength

	Quartiles of Prudent					
	Q1	Q2	Q3	Q4	p for trend	p for interaction
Gender						0.874
Male	0.00	0.73 (-1.03 to 2.50)	1.31 (-0.52 to 3.14)	1.44 (-0.40 to 3.28)	0.100	
Female	0.00	1.06 (0.18 to 1.94)	0.88 (0.01 to 1.76)	0.91 (0.02 to 1.80)	0.082	
Age ≥40 (years)						0.034
No	0.00	0.28 (-0.81 to 1.37)	1.25 (0.13 to 2.37)	1.49 (0.33 to 2.64)	0.003	
Yes	0.00	1.40 (-0.03 to 2.83)	0.49 (-0.92 to 1.90)	0.67 (-0.74 to 2.08)	0.849	
Smoking						0.339
Non	0.00	1.16 (0.16 to 2.16)	0.75 (-0.25 to 1.74)	0.95 (-0.07 to 1.97)	0.157	
Smoker	0.00	-0.12 (-2.29 to 2.04)	0.31 (-1.90 to 2.52)	1.49 (-0.69 to 3.66)	0.160	
Ex-smoker	0.00	0.97 (-1.53 to 3.48)	2.91 (0.35 to 5.47)	1.09 (-1.48 to 3.65)	0.196	
Leisure time PA (MET hours/week)						0.530
T1 (low)	0.00	0.10 (-1.19 to 1.39)	1.08 (-0.25 to 2.42)	1.32 (-0.04 to 2.68)	0.025	
T2	0.00	1.71 (0.05 to 3.38)	1.74 (0.10 to 3.37)	1.56 (-0.15 to 3.26)	0.091	
T3 (high)	0.00	1.46 (-0.19 to 3.11)	0.60 (-1.06 to 2.27)	0.78 (-0.86 to 2.42)	0.635	
Education						0.448
Low	0.00	0.26 (-2.27 to 2.79)	1.82 (-0.68 to 4.33)	1.95 (-0.55 to 4.46)	0.048	
Medium	0.00	1.15 (-0.36 to 2.66)	0.35 (-1.13 to 1.83)	-0.01 (-1.44 to 1.43)	0.786	
High	0.00	1.01 (-0.17 to 2.18)	1.18 (-0.04 to 2.40)	1.64 (0.36 to 2.92)	0.013	
Depression symptom						0.694
No	0.00	1.03 (-0.02 to 2.08)	1.01 (-0.05 to 2.07)	0.98 (-0.10 to 2.05)	0.116	
Yes	0.00	0.12 (-1.41 to 1.65)	0.90 (-0.66 to 2.46)	1.28 (-0.27 to 2.83)	0.072	
Hypertension						0.588
No	0.00	0.95 (0.04 to 1.86)	0.82 (-0.10 to 1.75)	1.06 (0.11 to 2.00)	0.045	
Yes	0.00	-0.47 (-3.42 to 2.48)	1.65 (-1.10 to 4.39)	1.03 (-1.63 to 3.68)	0.238	
On diet						0.287
No	0.00	0.60 (-0.34 to 1.54)	1.09 (0.13 to 2.06)	0.72 (-0.29 to 1.72)	0.087	

Yes	0.00	2.13 (0.01 to 4.24)	0.98 (-1.09 to 3.04)	1.65 (-0.33 to 3.63)	0.313	
Diabetes						0.631
No	0.00	1.04 (0.12 to 1.97)	1.11 (0.16 to 2.05)	1.19 (0.23 to 2.14)	0.018	
Yes	0.00	-0.01 (-2.37 to 2.36)	0.26 (-2.03 to 2.55)	0.52 (-1.81 to 2.85)	0.604	

Values were regression coefficients (95%CI) from linear regression. The p values for trend were tested using quartiles of dietary patterns as ordinal variables in the models. Model adjusted for age, sex, education, smoking, physical activity and diabetes.

Table S5 Subgroup analyses of the association between Prudent dietary pattern and relative handgrip strength

	Quartiles of Prudent					
	Q1	Q2	Q3	Q4	p for trend	p for interaction
Gender						0.765
Male	0.00	-0.01 (-0.08 to 0.07)	0.02 (-0.06 to 0.09)	0.04 (-0.04 to 0.11)	0.290	
Female	0.00	0.02 (-0.01 to 0.05)	0.02 (-0.01 to 0.06)	0.02 (-0.01 to 0.06)	0.182	
Age ≥40 (years)						0.402
No	0.00	0.00 (-0.04 to 0.05)	0.04 (-0.01 to 0.08)	0.04 (-0.01 to 0.09)	0.058	
Yes	0.00	-0.00 (-0.06 to 0.05)	-0.01 (-0.07 to 0.04)	0.00 (-0.05 to 0.05)	0.920	
Smoking						0.409
Non	0.00	0.01 (-0.03 to 0.05)	0.02 (-0.02 to 0.05)	0.03 (-0.01 to 0.07)	0.153	
Smoker	0.00	0.00 (-0.09 to 0.09)	-0.03 (-0.12 to 0.06)	-0.00 (-0.10 to 0.09)	0.761	
Ex-smoker	0.00	0.02 (-0.09 to 0.12)	0.10 (-0.00 to 0.20)	0.06 (-0.05 to 0.16)	0.119	
Leisure time PA (MET hours/week)						0.470
T1 (low)	0.00	-0.00 (-0.05 to 0.05)	0.02 (-0.03 to 0.08)	0.06 (0.01 to 0.12)	0.025	
T2	0.00	0.04 (-0.03 to 0.10)	0.06 (-0.00 to 0.13)	0.04 (-0.03 to 0.11)	0.187	
T3 (high)	0.00	0.00 (-0.07 to 0.07)	-0.01 (-0.08 to 0.06)	-0.01 (-0.08 to 0.05)	0.603	
Education						0.974
Low	0.00	-0.02 (-0.12 to 0.07)	0.04 (-0.05 to 0.13)	0.03 (-0.06 to 0.12)	0.233	
Medium	0.00	-0.01 (-0.07 to 0.06)	-0.00 (-0.06 to 0.06)	0.01 (-0.05 to 0.07)	0.764	
High	0.00	0.02 (-0.03 to 0.07)	0.02 (-0.03 to 0.07)	0.04 (-0.02 to 0.09)	0.174	
Depression symptom						0.579
No	0.00	0.00 (-0.04 to 0.05)	0.01 (-0.04 to 0.05)	0.02 (-0.03 to 0.06)	0.468	
Yes	0.00	-0.01 (-0.07 to 0.06)	0.04 (-0.03 to 0.10)	0.05 (-0.01 to 0.11)	0.075	
Hypertension						0.607
No	0.00	0.01 (-0.03 to 0.05)	0.02 (-0.02 to 0.06)	0.04 (-0.00 to 0.08)	0.063	
Yes	0.00	-0.07 (-0.17 to 0.03)	-0.02 (-0.11 to 0.07)	-0.04 (-0.13 to 0.05)	0.760	
On diet						0.648
No	0.00	-0.00 (-0.04 to 0.04)	0.02 (-0.02 to 0.06)	0.02 (-0.02 to 0.06)	0.177	

Yes	0.00	0.05 (-0.03 to 0.14)	0.03 (-0.05 to 0.11)	0.03 (-0.05 to 0.11)	0.621	
Diabetes						0.900
No	0.00	0.01 (-0.03 to 0.05)	0.03 (-0.01 to 0.06)	0.04 (-0.00 to 0.08)	0.053	
Yes	0.00	-0.01 (-0.09 to 0.08)	-0.01 (-0.10 to 0.07)	-0.01 (-0.10 to 0.08)	0.817	

Values were regression coefficients (95%CI) from linear regression. The p values for trend were tested using quartiles of dietary patterns as ordinal variables in the models. Model adjusted for age, sex, education, smoking, physical activity and diabetes.

Table S6 Subgroup analyses of the association between quartiles of weight loss and handgrip strength

	Quartiles of weight loss					
	Q1	Q2	Q3	Q4	p for trend	p for interaction
Gender						0.947
Male	0.00	0.37 (-2.02 to 2.75)	-0.72 (-2.84 to 1.40)	-1.46 (-3.44 to 0.51)	0.069	
Female	0.00	-0.21 (-1.04 to 0.61)	-0.79 (-1.66 to 0.09)	-1.00 (-2.08 to 0.07)	0.027	
Age ≥40 (years)						0.668
No	0.00	-0.57 (-1.89 to 0.75)	-1.33 (-2.57 to -0.09)	-1.75 (-3.01 to -0.49)	0.004	
Yes	0.00	0.06 (-1.17 to 1.28)	-0.35 (-1.68 to 0.98)	-0.72 (-2.28 to 0.84)	0.326	
Smoking						0.673
Non	0.00	-0.05 (-0.99 to 0.89)	-1.02 (-2.00 to -0.04)	-1.21 (-2.36 to -0.06)	0.010	
Smoker	0.00	1.10 (-1.90 to 4.09)	0.63 (-2.10 to 3.35)	-0.84 (-3.37 to 1.69)	0.259	
Ex-smoker	0.00	-1.77 (-4.66 to 1.13)	-1.66 (-4.50 to 1.18)	-1.65 (-4.48 to 1.18)	0.300	
Leisure time PA (MET hours/week)						0.695
T1 (low)	0.00	-0.35 (-1.67 to 0.96)	-1.12 (-2.47 to 0.23)	-1.31 (-2.79 to 0.17)	0.044	
T2	0.00	0.65 (-1.02 to 2.32)	-0.28 (-1.96 to 1.41)	-2.19 (-4.06 to -0.31)	0.028	
T3 (high)	0.00	-0.56 (-2.39 to 1.26)	-0.76 (-2.55 to 1.04)	-0.70 (-2.52 to 1.11)	0.450	
Education						0.357
Low	0.00	-1.93 (-4.03 to 0.17)	-1.11 (-3.41 to 1.18)	-3.36 (-6.02 to -0.71)	0.039	
Medium	0.00	1.02 (-0.65 to 2.68)	0.19 (-1.40 to 1.79)	0.07 (-1.60 to 1.73)	0.871	
High	0.00	-0.20 (-1.44 to 1.05)	-1.27 (-2.54 to -0.00)	-1.86 (-3.22 to -0.49)	0.003	
Depression symptom						0.993
No	0.00	-0.08 (-1.13 to 0.97)	-0.84 (-1.92 to 0.24)	-1.45 (-2.63 to -0.28)	0.008	
Yes	0.00	-0.42 (-2.16 to 1.32)	-0.74 (-2.38 to 0.91)	-0.89 (-2.61 to 0.82)	0.280	
Hypertension						0.901
No	0.00	-0.15 (-1.13 to 0.83)	-0.90 (-1.87 to 0.06)	-1.37 (-2.39 to -0.35)	0.004	
Yes	0.00	-0.61 (-2.99 to 1.77)	-1.05 (-3.86 to 1.76)	-0.59 (-3.78 to 2.60)	0.570	
On diet						0.398
No	0.00	-0.43 (-1.45 to 0.59)	-0.78 (-1.79 to 0.24)	-1.29 (-2.39 to -0.20)	0.018	

Yes	0.00	0.85 (-1.09 to 2.79)	-0.81 (-2.81 to 1.20)	-1.36 (-3.45 to 0.72)	0.125	
Diabetes						0.876
No	0.00	-0.17 (-1.17 to 0.82)	-0.81 (-1.80 to 0.17)	-1.22 (-2.26 to -0.19)	0.012	
Yes	0.00	0.04 (-2.16 to 2.24)	-0.88 (-3.22 to 1.46)	-1.97 (-4.67 to 0.74)	0.132	

Values were regression coefficients (95%CI) from linear regression. p values for trend were tested using quartiles of dietary patterns as ordinal variables in the models. Model adjusted for age, sex, education, smoking, physical activity and diabetes.

Table S7 Subgroup analyses of the association between quartiles of weight loss and relative handgrip strength

	Quartiles of weight loss					
	Q1	Q2	Q3	Q4	p for trend	p for interaction
Gender						0.019
Male	0.00	0.06 (-0.04 to 0.15)	0.10 (0.01 to 0.18)	0.15 (0.07 to 0.23)	<0.001	
Female	0.00	0.04 (0.01 to 0.07)	0.05 (0.02 to 0.08)	0.05 (0.01 to 0.09)	0.003	
Age ≥40 (years)						0.004
No	0.00	0.06 (0.01 to 0.11)	0.05 (0.00 to 0.10)	0.13 (0.08 to 0.18)	<0.001	
Yes	0.00	0.03 (-0.02 to 0.07)	0.08 (0.03 to 0.13)	0.02 (-0.04 to 0.08)	0.068	
Smoking						0.117
Non	0.00	0.03 (-0.01 to 0.06)	0.04 (0.01 to 0.08)	0.06 (0.02 to 0.10)	0.003	
Smoker	0.00	0.13 (0.01 to 0.25)	0.15 (0.04 to 0.26)	0.17 (0.07 to 0.28)	0.002	
Ex-smoker	0.00	0.05 (-0.06 to 0.16)	0.06 (-0.05 to 0.17)	0.12 (0.00 to 0.23)	0.043	
Leisure time PA (MET hours/week)						0.065
T1 (low)	0.00	0.03 (-0.02 to 0.08)	0.05 (-0.00 to 0.10)	0.09 (0.04 to 0.15)	0.002	
T2	0.00	0.08 (0.02 to 0.15)	0.10 (0.03 to 0.16)	0.04 (-0.03 to 0.11)	0.108	
T3 (high)	0.00	0.02 (-0.05 to 0.09)	0.04 (-0.03 to 0.11)	0.12 (0.05 to 0.20)	<0.001	
Education						0.115
Low	0.00	-0.01 (-0.09 to 0.06)	0.00 (-0.08 to 0.08)	0.01 (-0.08 to 0.10)	0.728	
Medium	0.00	0.07 (0.01 to 0.13)	0.11 (0.05 to 0.17)	0.18 (0.12 to 0.24)	<0.001	
High	0.00	0.04 (-0.01 to 0.10)	0.04 (-0.01 to 0.10)	0.06 (0.00 to 0.11)	0.053	
Depression symptom						0.996
No	0.00	0.04 (-0.00 to 0.08)	0.06 (0.02 to 0.10)	0.10 (0.05 to 0.14)	<0.001	
Yes	0.00	0.04 (-0.03 to 0.11)	0.07 (0.00 to 0.13)	0.10 (0.03 to 0.17)	0.005	
Hypertension						0.508
No	0.00	0.05 (0.01 to 0.09)	0.06 (0.02 to 0.10)	0.10 (0.06 to 0.14)	<0.001	
Yes	0.00	-0.02 (-0.10 to 0.06)	0.05 (-0.04 to 0.15)	0.07 (-0.04 to 0.17)	0.129	
On diet						0.068
No	0.00	0.04 (0.00 to 0.08)	0.08 (0.04 to 0.12)	0.11 (0.07 to 0.16)	<0.001	

Yes	0.00	0.05 (-0.03 to 0.12)	-0.01 (-0.08 to 0.07)	0.04 (-0.04 to 0.12)	0.582	
Diabetes						0.026
No	0.00	0.06 (0.02 to 0.10)	0.06 (0.02 to 0.10)	0.11 (0.07 to 0.15)	<0.001	
Yes	0.00	0.01 (-0.07 to 0.09)	0.08 (-0.01 to 0.16)	0.01 (-0.09 to 0.11)	0.386	

Values were regression coefficients (95%CI) from linear regression. p values for trend were tested using quartiles of dietary patterns as ordinal variables in the models. Model adjusted for age, sex, education, smoking, physical activity and diabetes.
